# Magnitude and Determinants of Antenatal Care Utilization in Kandahar City, Afghanistan

**DOI:** 10.1155/2021/5201682

**Published:** 2021-07-02

**Authors:** Muhammad Haroon Stanikzai, Mohammad Hashim Wafa, Abdul Wahed Wasiq, Hadia Sayam

**Affiliations:** ^1^Public Health Department, Faculty of Medicine, Kandahar University, Kandahar, Afghanistan; ^2^Neuropsychiatric and Behavioral Science Department, Faculty of Medicine, Kandahar University, Kandahar, Afghanistan; ^3^Internal Medicine Department, Faculty of Medicine, Kandahar University, Kandahar, Afghanistan; ^4^Para-Clinic Department, Faculty of Medicine, Malalay Institute of Higher Education, Kandahar, Afghanistan

## Abstract

**Background:**

Women's and children's health is a crucial public health concern that epitomizes the universal platform for Sustainable Development Goals (SDGs). Appropriate and timely care during pregnancy can improve maternal and child health.

**Objectives:**

The present study aimed at determining the magnitude and determinants of antenatal care services' utilization in Kandahar city.

**Methods:**

A community-based cross-sectional study involving 850 women with at least one delivery in the last 2 years was carried out in Kandahar city from January to February 2021. Questionnaires to record information on sociodemographic, reproductive, and antenatal care- (ANC-) related characteristics were administered. Data were analyzed using SPSS 21.00 statistical software. We used descriptive statistics such as frequency and percentages to present the data. Determinants of antenatal care services' utilization were determined using a multivariable logistic regression model.

**Results:**

Among all study participants, 589 (69.3%, 95% confidence interval (CI) = 66.0%–72.4%) of study participants utilized antenatal care services at least once. However, only 22% of the women were utilizing the recommended ≥4 ANC visits. Factors that remained significantly associated with antenatal care services' utilization in multivariable analysis included women's educational status (adjusted odds ratio (AOR) = 2.0, 95% CI: 1.0–4.3), pregnancy intention (AOR = 2.1, 95% CI: 1.1–3.4), and place of residence (AOR = 1.7, 95% CI: 1.1–2.6).

**Conclusion:**

This study has found high rates (vs. the national level) of antenatal care services' utilization among women who had at least one delivery in the last 2 years. However, the rate of recommended ≥4 ANC visits was low. Factors determining antenatal care utilization such as educational status of the mother, pregnancy intention, and place of residence hold the key to address the issue of ANC services lower utilization and consequently improve maternal and fetal health.

## 1. Introduction

Women's and children's health is a crucial public health concern that epitomizes the universal platform for Sustainable Development Goals (SDGs). Only in 2017, for instance, 295000 cases of maternal deaths were reported globally. It is asserted that maternal mortality figures pertain predominantly to low- and middle-income countries [1].

There is a myriad of well-established and cost-effective interventions, such as antenatal care (ANC), skilled attendant delivery, and postnatal care (PNC) that reduce maternal mortality. ANC entails specialized professional care offered to pregnant women from the onset of pregnancy until delivery. Its utilization ensures the prevention of maternal health risks, safe delivery, and good health of the newborn. It is therefore considered one of the four key strategies that connote reduction in maternal and childhood morbidity and mortality. World Health Organization (WHO) recommends a minimum of eight ANC contacts with the first visit during the first trimester of gestational age [2].

The proportion of women utilizing ANC services reflects discrepancies in different parts of the world. Moreover, ANC application is suboptimal, particularly in the developing world [3]. Data assert that about 98% of pregnant women receive ANC at least once in the developed world [3]. Of them, 81.9% initiate their ANC at the recommended time [4]. In the developing countries, however, it is as low as 68% (24% early ANC) [4]. South Asia holds the lowest (54%) ANC record of at least one ANC visit per pregnancy [3]. Therefore, it signifies a salient instance of a low-income region of the world. Likewise, Afghanistan Demographic Health Survey 2015 (ADHS, 2015) found that 59% of pregnant women attended ANC visits at least once, while only 18% claimed four or more ANC visits [5, 6].

Perpetual findings of extensive research conducted in the developing world indicate that diverse factors such as maternal age [7, 8], maternal education [6, 8–10], household income [8, 10], husband education [8, 11], maternal employment [12, 13], area of residence [8, 10, 14], pregnancy intention [15, 16], media exposure [16–18], pregnancy-linked cost [19, 20], history of obstetric complications [11, 21], parity [11, 22], lack of ANC knowledge [23–25], and the distance from a health facility [25, 26] are associated with ANC utilization.

It is evident from ADHS (2015) that there was significant variation in antenatal care utilization by province. Women in the south were the lowest user of antenatal care services. Therefore, we sought to investigate the magnitude of antenatal care utilization and its determinants in Kandahar city. The present study would help policymakers to improve their knowledge regarding determinants of antenatal care utilization and subsequently can develop relevant policies for higher utilization of antenatal care services in the south.

## 2. Materials and Methods

### 2.1. Study Setting

This cross-sectional household survey was carried out in Kandahar city from January to February 2021. Kandahar is located in the southwest and is considered the second-largest city in Afghanistan. The city is divided into fifteen administrative units or districts and is lodging approximately 512000 people. In terms of public health service provision, there is a myriad of Primary Healthcare Clinics (PHCs), thirteen Comprehensive Health Clinics (CHCs), and two regional hospitals with secondary and tertiary healthcare capacities.

For data collection, we randomly selected four districts (4, 7, 12, 14) from the list of fifteen districts. A district typically lodges 7000–9000 households. The households within every district are numbered. The data were collected from 850 households.

### 2.2. Study Design

We employed a cross-sectional survey design, using quantitative data collection methods and statistical analysis. As this study is aimed at determining the determinants of ANC utilization, a cross-sectional design was appropriate because this design allows researchers to collect data on respondents' characteristics and information about the outcome simultaneously [18–22]. Furthermore, it determines the relationship between the independent characteristics of respondents with the outcome of interest.

### 2.3. Sample Size and Sampling Procedures

We employed single population proportion formula [*n* = *Z*^2^*P* (1−*P*)/(*d*)^2^] for calculating our sample size. Our postulation of 95% confidence interval, 5% margin of error, *p*=0.51 [5] (the proportion of ANC services utilization in Kandahar), 10% nonresponse rate, and a design effect of 2 resulted in a sample size of 850 households.

To pick a sample of households per district, we used stratified systematic random sampling. The number of selected households was proportionate to the household density of that district. The sampling interval (*k*) for every district was extracted from the ratio of district households to the sample size. To select a starting household, we picked a random number from 1 to *k*, and thus every *k*th household was included.

### 2.4. Inclusion and Exclusion Criteria

Participants with a history of at least one delivery in the last two years were eligible.

Unwillingness to participate and those who presented with a critical condition composed our exclusion criteria.

### 2.5. Study Variables

This study employed ANC service utilization status as a dependent variable. The outcome variable was binary and it was coded as 1 if the women were utilizing ANC services and 0 if the women were not utilizing ANC services. Our independent variables are well-documented and they consist of maternal age, educational level, employment status, residential area, income, pregnancy intention, history of obstetric complications, parity, birth order of the last child, and distance from health facility.

### 2.6. Data Collection

We used a structured and pretested questionnaire to collect the data. The study instruments were initially prepared in English and translated into Pashto and back into English to ensure that the meaning of questions is preserved. Prior to the commencement of the study, it was pilot tested on 5% of the total sample in another setting (Aino Mena, Kandahar city). The recruiters collected data pertinent to subjects' sociodemographic, reproductive, and ANC utilization status.

The data were collected by three pairs of nurses (one male and one female) and one supervisor (health professional). Prior to the pilot study the principal investigators organized a two-day training session for the recruiters. The objective was to enhance their skills of sampling, interviewing, filling out the questionnaires, and addressing potential ethical concerns that may emerge during the study. The quality of the collected data was assessed by the principal investigators through a random survey of the households. On a daily basis, the questionnaires were checked for completion.

### 2.7. Statistical Analysis

All questionnaires were first coded and entered into Microsoft Excel (2019) and later exported into IBM SPSS version 21 for data cleaning and analysis [14]. We used a binary logistic regression model to assess the determinant of antennal natal care utilization. Variables with *p* value of less than 0.25 were retained into multivariable logistic regression. Finally, multivariable logistics analysis with enter method was carried out to determine independent determinants of ANC utilization. The two-tailed *p* values of <0.05 were considered statistically significant.

### 2.8. Ethical Consideration

This study received ethical clearance from the Research and Ethics Committee of Kandahar University (Makotob no. 53, dated on 28/7/2019). We obtained the permission of data collection from Kandahar Municipality. Additionally, the study was explained to every potential participant and informed verbal consents were obtained from all subjects.

## 3. Results

### 3.1. Sociodemographic Characteristics of the Subjects

The mean age of our subjects was 28 (±7.1). The vast majority of the subjects (694, 82%) were from the age group 20–35, 143 (16.8%) were from the age group > 35, and 13 (1.5%) were from the age group < 20. As portrayed in Table 1, most of the respondents (94.1%) had no formal education, 24 (2.8%) had primary schooling, and only 18 (2.1%) had attained religious education. Almost all (99.5%) women were housewives and unemployed. Of their husbands' education and occupation, approximately 86.1% (732) of them had no formal education and about 93.3% (793) were self-employed. Moreover, 81.5% (693) of the subjects' families were living within *a* < 5 km distance of a health facility and more than half (64%) of the families were having more than twelve members. In terms of their monthly household income, 516 (60.7%) families of our subjects had a monthly household income of Afghanis 5000–10000, and the rest (334; 39.3%) gained > Afghanis 10000 per month (Table 1).

### 3.2. Reproductive Characteristics of the Study Participants

We included a total of 850 women who delivered at least one viable child within the last two years. Of them, 770 (90.6%) were multiparous. A minority of our subjects (158; 18.6%) have used diverse contraceptives before their last conception. Of them, 86 (10.1%) used oral contraceptive pills and 44 (5.2%) used male condoms. A staggering number of the subjects (760; 89.4%) have planned their last pregnancies and 610 (71.8%) have had experienced delivery in the hospital. Around 46.1% (392) of the women encountered complications during their last pregnancies.  Table 2 depicts the characteristics of our subjects' reproductive variables.

### 3.3. Utilization of Antenatal Care Services

A total of 589 subjects (69.3%, 95% CI = 66.07%–72.38%) utilized antenatal care services. Of these 336 (57%, 95% CI = 52.9%–61.08%) presented early (<12 weeks) and 253 (43%, 95% CI = 38.9%–47.06%) presented late (>12 weeks) for their first ANC visit. Our results reveal that only 128 (21.7%) mothers asserted the recommended (≥4) visits of the antenatal care services. Nearly 59% (347) of our study participants visited Comprehensive Health Clinics (CHCs), 231 (39.3%) visited hospitals, and 11 (1.8%) visited private clinics for their antenatal care services. The majority of the subjects who utilized antenatal care services (528; 89.6%) received health education in each ANC visit and 483 (82%) believed that ANC is important for the health of both the mother and the child. Among 261 nonutilizers, common reasons for not utilizing ANC were family problems (125, 47.9%), transportation problems (67, 25.7%), and lack of awareness (57, 21.8%) (Table 3).

### 3.4. Determinants of Antenatal Care Services' Utilization

The multiple logistic regression showed that the odds of utilizing antenatal care services were higher in educated women (AOR = 2.09, 95% CI: 1.00–4.37) as compared to women with no formal education. Besides, women who planned their last pregnancy had 2.16 times higher odds of utilizing antenatal care services (AOR = 2.16, 95% CI: 1.14–3.40) than the women with an unplanned pregnancy. Furthermore, in contrast to the women living in the twelfth district the expected number of antenatal care utilization visits for the women who were living in the seventh district was 1.72 times higher (AOR = 1.72, 95% CI: 1.10–2.67).  Table 4 summarizes the results of the bivariate and multivariable analysis.

## 4. Discussion

This is the first study, of which we are aware, to determine the magnitude of ANC services utilization and its determinants among women who had at least one delivery in the last two years in Kandahar city. The results of this community-based cross-sectional study were consistent with the findings of other pertinent studies conducted in developing countries [7–11, 18, 23]. We found that ANC utilization was highly associated with the educational status of the mother, pregnancy intention, and her place of residence. Our findings showed that these factors should be considered in future programs to enhance the utilization of antenatal care services.

We found that 69.3% of our subjects (the mothers) made at least one ANC visit with a 95% CI of 66.07% to 72.38%. This figure is higher than the national level (59%) reported by ADHS in 2015 [5, 6] and comparable with another study conducted in Kandahar province [27]. The rate of ANC utilization in the present study is consistent with those asserted in many studies conducted in other developing countries, such as Rwanda, Bangladesh, Jordan, and Ethiopia [7–11, 18, 23]. This finding epitomizes many developing countries where a substantial proportion of pregnant women fail to regularly make their ANC visits. ANC utilization rates, however, have been shown higher in the developed world probably due to greater access and awareness as well as other sociodemographic disparities between the two settings [4].

We noticed that out of 589 mothers who did visit ANC services only 22% of them have made the recommended ≥4 ANC visits. This finding of low utilization is of great importance. It suggests that while a significant number of the mothers (69.3%) were aware of ANC services, a vast majority did not succeed to make the recommended ≥ 4 ANC visits. Hence, further studies are warranted to address what brings about such low utilization of ANC services.

Our data indicate a fairly strong association between women' educational status and the utilization of ANC services. Women who had basic, secondary, and tertiary education were more likely to utilize ANC services in comparison to those who had no formal education. This finding is consistent with previous studies from other developing countries [6, 8–10]. One solution is to strengthen and expand primary education and female literacy programs to improve women's health in the long term [5]. Other approaches include health educational programs and public awareness campaigns to be undertaken in the light of low health literacy on the utilization of antenatal care services in Kandahar province.

Additionally, we observed that pregnancy intention was a significant determinant of ANC services utilization. Women with planned pregnancies were about two times more likely to utilize ANC than those with unplanned pregnancies. Studies conducted in Ethiopia, Brazil, India, and other developing countries reflect a comparable finding [15, 28–30]. Given this finding, it is imperative to increase the uptake of family planning services that will promote planned pregnancies and can subsequently encourage the utilization of antenatal care services [31].

As our third important finding, and consistent with other studies conducted in Bangladesh [8] and Ethiopia [10], the area of residence of the mothers was strongly associated with ANC utilization; e.g., women living in the seventh district of Kandahar city were about two times more likely to utilize ANC services than those who lived in the twelfth district. This higher rate of ANC utilization among the residents of the seventh district probably reflects the value of living in the close vicinity of a health facility. It is also argued that there might be other system-related factors, such as home visits and more health facilities, that may have influenced ANC services utilization in this district. Hence, addressing the aforementioned barriers holds the key to improving antenatal care utilization in these settings.

## 5. Strengths and Limitations

Although this study found some crucial factors that were significantly associated with ANC utilization by pregnant women, our findings are to be interpreted in light of the following limitations. First, the cross-sectional nature of the study limits the temporal relationship between the variables. Secondly, insecurity of our study area and the poverty of the subjects may have somehow influenced our results. Furthermore, we defined place of residence as the respondent's residential area during the data collection period, but antenatal care utilization was assessed over the last two years and therefore there might be a measurement bias. Finally, our data were collected in the metropolitan area. Therefore, its generalizability is limited in terms of not representing the rural population and nomads. We suggest that further in-depth investigation of health system-related factors associated with ANC utilization is highly needed to be conducted on provincial and country levels.

## 6. Conclusion

In this community-based study, we found that more than half (69.3%) of our subjects utilized ANC services at least once. However, only 22% of the women were utilizing the recommended ≥4 ANC visits. Factors determining antenatal care utilization such as educational status of the mother, pregnancy intention, and place of residence hold the key to address the issue of ANC services lower utilization and consequently improve maternal and fetal health.

## 7. Implications for Practice and/or Policy

This study provides information on the determinants of antenatal care utilization in Kandahar city. Policymakers and program planners should be aware that factors such as the education status of the women, pregnancy intention, and place of residence are associated with a greater chance of ANC utilization. Limited information is available on the health-system-related factors that influence ANC utilization. We recommend further in-depth investigation of health system-related factors associated with ANC utilization on provincial and country levels.

## Figures and Tables

**Table 1:** Sociodemographic characteristics of study participants (*n* = 850).

Variable	Frequency (%)
*Age groups*	
<20	13 (1.5)
20–35	694 (81.6)
>35	143 (16.8)

*Districts*	
Fourth	240 (28.2)
Seventh	180 (21.2)
Twelfth	260 (30.6)
Fourteenth	170 (20.0)

*Language spoken*	
Pashto	801 (94.2)
Dari	49 (5.8)

*Women educational status*	
No formal education	800 (94.1)
Primary	24 (2.8)
Secondary	6 (0.7)
High school graduate	2 (0.2)
Religious (Madrasa)	18 (2.1)

*Husband education*	
No formal education	732 (86.1)
Primary	30 (3.5)
Secondary	24 (2.8)
High school graduate	23 (2.7)
Higher education	13 (1.5)
Religious (Madrasa)	28 (3.3)

*Women occupation*	
Unemployed/housewife	846 (99.5)
Government employed	2 (0.2)
Self-employed	2 (0.2)

*Husband occupation*	
Government employed	17 (2)
Self-employed	793 (93.3)
Unemployed	40 (4.7)

*House distance from the health facility*	
≤5 km	693 (81.5)
>5 km	157 (18.5)

*Family size*	
1 to 7 people	31 (3.6)
8 to 12 people	275 (32.4)
>12 people	544 (64)

*Household income (Afghanis)*	
5000–10000	516 (60.7)
>10000	334 (39.3)

**Table 2:** Reproductive characteristics of the study population.

Variable	Frequency (%)
*Parity (n* *=* *850)*	
Primiparous	80 (9.4)
Multiparous	770 (90.6)

*Birth order of the last child (n* *=* *850)*	
1–3	509 (59.9)
4–6	231 (27.2)
≥7	110 (12.9)

*Contraceptive use in the past (n* *=* *850)*	
Have not used	692 (81.4)
Male condom	44 (5.2)
Injectables	16 (1.9)
Oral contraceptives	86 (10.1)
IUD	12 (1.4)

*Previous pregnancy planned (n* *=* *850)*	
Yes	760 (89.4)
No	90 (10.6)

*Place of last delivery (n* *=* *850)*	
Health facility	610 (71.8)
Home	240 (28.2)

*History of complications in last pregnancy (n* *=* *850)*	
Yes	392 (46.1)
No	458 (53.9)

**Table 3:** Antenatal care utilization and its related characteristics.

Variable	Frequency (%)
*ANC visits (n* *=* *850)*	
Yes	589 (69.3)
No	261 (30.7)

*Trimester of ANC visits (n* *=* *589)*	
First	336 (57)
Second	199 (33.8)
Third	54 (9.2)

*Total number of ANC visits (n* *=* *589)*	
1	243 (41.2)
2	218 (37.1)
4 and above	128 (21.7)

*Place for ANC visits (n* *=* *589)*	
Comprehensive health clinic	347 (58.9)
Hospitals	231(39.3)
Private clinic	11 (1.8)

*ANC services' awareness (n* *=* *589)*	
Health institutions	153 (25.9)
Media	108 (18.4)
Family and friends	328 (55.7)

*Who benefited the most from ANC visits (n* *=* *589)*	
Mother	18 (3.1)
Child	88 (14.9)
Both	483 (82)

*Health education received in each ANC visit (n* *=* *589)*	
Yes	528 (89.6)
No	46 (7.8)
Do not know	15 (2.6)

*Behaviors of the ANC provider (n* *=* *589)*	
Satisfactory	413 (70.1)
Unsatisfactory	176 (29.9)

*Reasons for not visiting the ANC facility (n* *=* *261)*	
Lack of awareness	57 (21.8)
Transportation problem	67 (25.7)
Family problems	125 (47.9)
Personal problems	12 (4.6)

**Table 4:** Determinants of antenatal care utilization in Kandahar city, 2021: crude and adjusted odds ratio.

Independent variable	Categories	Crude odds ratio (95% CI)	*p* value	Adjusted odds ratio (95% CI)	*p* value
Women education	Educated	3.09 (2.00–4.37)	0.045	2.09 (1.00–4.37)	0.017
	Uneducated	1		1	

Previous pregnancy	Planned	2.16 (1.38–3.36)	0.001	2.10 (1.14–3.40)	0.014
	Unplanned	1		1	

District	Twelve	1	0.003	1	0.017
	Seven	1.92 (1.24–2.95)	0.29	1.72 (1.10–2.67)	
	Four	1.22 (0.84–1.77)	0.14	—	
	Fourteen	0.37 (0.48–1.11)		—	

## Data Availability

The primary data used to support the findings of this study are available from the corresponding author upon request.
